# Molecular diagnostics of neurodegenerative disorders

**DOI:** 10.3389/fmolb.2015.00054

**Published:** 2015-09-22

**Authors:** Megha Agrawal, Abhijit Biswas

**Affiliations:** ^1^Department of Biology, University of Arkansas at Little RockLittle Rock, AR, USA; ^2^Department of Electrical Engineering, Center for Nano Science and Technology, University of Notre DameNotre Dame, IN, USA

**Keywords:** biomarkers, miRNAs, Alzheimer's disease, Parkinson's disease, Amyotrophic lateral sclerosis, Huntington's disease, neurodegeneration, neurons

## Abstract

Molecular diagnostics provide a powerful method to detect and diagnose various neurological diseases such as Alzheimer's and Parkinson's disease. The confirmation of such diagnosis allows early detection and subsequent medical counseling that help specific patients to undergo clinically important drug trials. This provides a medical pathway to have better insight of neurogenesis and eventual cure of the neurodegenerative diseases. In this short review, we present recent advances in molecular diagnostics especially biomarkers and imaging spectroscopy for neurological diseases. We describe advances made in Alzheimer's disease (AD), Parkinson's disease (PD), Amyotrophic lateral sclerosis (ALS) and Huntington's disease (HD), and finally present a perspective on the future directions to provide a framework for further developments and refinements of molecular diagnostics to combat neurodegenerative disorders.

## Introduction

Neurodegenerative disorders correspond to the disorders in the central nervous system that are characterized by the progressive loss of neural tissues. Changes in the neurons cause them to function abnormally and eventually result in the cells' demise. The reason is the inability of the neurons to regenerate on their own after the neural deterioration or severe damage. At present, roughly around 5 million Americans suffer from Alzheimer's disease (AD); 1 million from Parkinson's disease (PD); 400,000 from multiple sclerosis (MS); 30,000 from Amyotrophic lateral sclerosis (ALS), and 3000 from Huntington's disease (HD). The incidence is expected to soar as the population ages, because neurodegenerative diseases strike primarily in mid-to late-life. Neuroregeneration is a viable way to curb neurodenegerative disorders. One of the current approaches is stem cell therapy that has shown to be potentially helpful in neuroregeneration or even neuronal cell replacement (Chung et al., 2002; Rachakonda et al., 2004).

An early detection of the onset of neurodegeneration is vital as it can provide a chance for an early treatment that may be helpful to prevent further progression of the disease. Among current diagnostics, neuropathology is considered as the gold standard (Chung et al., 2002). However, it is usually based on an autopsy that is done after the death of a patient. Therefore, medical researchers are in search for an effective non-invasive diagnostic method that can be employed for an early detection of neurodegeneration when a pharmacological intervention is still possible.

Molecular diagnosis has emerged as a powerful technique that can be helpful for an early detection of various neurodegenerative disorders. One of the powerful molecular diagnostics is the application of biomarkers. Biomarkers are basically biological molecular substances that are used to indicate the presence or onset of a certain disorder. Normal and abnormal biological processes can be detected by the use of biomarkers. The principal requirement for a good biomarker is its preciseness and reliability. It should also be able to distinguish between the healthy and the diseased tissues, and should differentiate between different diseases. Biomarkers are considered promising in aiding in early diagnosis and setting standards for the development of new remedies to treat neuronal disorders (Chung et al., 2002; Peterson, 2002; Rachakonda et al., 2004). Biomarkers may be measured using imaging techniques such as positron emission tomography (PET), magnetic resonance imaging (MRI), and nuclear magnetic resonance spectroscopy (NMRS). Table 1 summarizes various molecular diagnostic markers for neurodegenerative diseases.

**Table 1:** **A summary giving genetic and biochemical diagnostic markers for Alzheimer's, Parkinson's, Amyotrophic lateral sclerosis, and Huntington's Disease**.

**Diseases**	**Genetic diagnostic markers**	**Biochemical diagnostic markers**
Alzheimer's Disease (AD)	Amyloid precursor protein mutations	Plasma/CSF Aβ_1−42_ peptide
	Presinilin-1 gene mutations	CSF tau protein
	Presinilin-2 gene mutations	Phospho-tau
	*ApoE* isoforms	
	*ApoE*polymormisms	
Parkinson's Disease (PD)	α-synuclein gene mutations	Loss of Dopamine transporter (DAT)
	Parkin gene mutations	Lewy bodies
	*UCH-L1* gene mutations	
	*PINK1* gene mutations	
	*DJ-1* gene mutations	
	*NR4A2* gene mutations	
Amyotrophic Lateral Sclerosis (ALS)	*ALS2* gene mutations	mGLUR2
	*NEFH* gene mutations	SOD1
	*SOD1* gene mutation	Glutathione
	*C9orf72* gene mutation	8OH2′dG
	*FUS* gene mutation	Cytokines
	*TARDBP* gene mutations	
Huntington's Disease (HD)	*HTT* gene mutations	Growth hormones
		Cytokines
		mGLUR2
		SOD1
		Glutathione
		8OH2′dG

Among other recently developed molecular diagnostics, biomarkers coupled with magnetic resonance spectroscopy (MRS) has shown promise. It is a quantitative imaging technique that allows *in-vivo* measurement of certain neuronal metabolites as biomarkers that can be used to study metabolic dysfunctions and irreversible neuronal damage (Ciurleo et al., 2014). The potential role of MRS as an *in-vivo* molecular imaging biomarker was investigated for early diagnosis of PD and for monitoring the efficacy of therapeutic interventions (Ciurleo et al., 2014).

Altered expression of microRNAs (miRNAs) in many disease states, including neurodegeneration along with applications of miRNAs in biological fluids in different pathologies make them promising candidates as neurodegenerative disease biomarkers that may lead to identify new therapeutic targets (Grasso et al., 2014). The role of miRNAs in the pathogenesis of neurodegeneration was investigated to gain insights into the possibilities and challenges of using these small RNA molecules as a signature for neurodegenerative conditions (Grasso et al., 2014). It is known that miRNAs can be transported by exosomes which are small membrane-derived vesicles secreted by many cell types, including neurons, astrocytes, oligodendrocytes, and microglia (Lachenal et al., 2011; Russo et al., 2012). Exosomes shed from stimulated blood cells and the vascular endothelium are involved in neurological disorders (Soreq et al., 2013). Exosomes have significant potential as biomarkers for disease diagnosis, as their molecular composition reflects the physiological or pathophysiological changes in their cell of origin (Lin et al., 2015). Furthermore, they can be isolated from biofluids such as blood and urine, making them very attractive targets for diagnostic application. It has been reported that exosomal amyloid peptides accumulate in the brain plaques of AD patients (Rajendran et al., 2006) and tau phosphorylated at Thr-181, an established biomarker for AD, is present at elevated levels in exosomes isolated from cerebrospinal fluid specimens of AD patients with mild symptoms (Saman et al., 2012). Studies have also shown release of α-synuclein in exosomes in an *in vitro* model system of PD (Alvarez-Erviti et al., 2011). These exosomal proteins may have great potential in clinical diagnostics and should be further explored, as the concept is still new in the biomarker discovery arena (Miller and O'Callaghan, 2015).

The research field of molecular diagnostics in neurodegenerative disorder is still a nascent area of research and development. It is anticipated that further developments in various molecular diagnostics would pave the way for the early detection and effective treatment of neurodegeneration. In this short review, we focus on recent advances made in major neurodegenerative disorders—AD, PD, ALS, and HD and the applications of molecular diagnosis for the potential remedies. We also present a perspective on the future direction of diagnostics and curbing the progression of neuronal disorders.

## Molecular diagnosis for neurodegenerative diseases: A potentially better approach over behavioral symptoms based diagnosis

Behavioral symptoms can be utilized for the pre-mortem diagnosis of neurodegenerative disorders. However, the major drawback of behavioral symptoms based diagnosis is its limitations to identify patients early in the course of their disease, when the pharmacological intervention can significantly prevent further progression of the disease, if detected early. For example, well-established behavioral tests like the ADAS-Cog that are regarded as the “gold-standard” for AD diagnosis may give false-negative results for patients with mild symptoms (Posner et al., 2013).

To overcome these diagnostics challenges, current neuro-pathologic methods have been combined with molecular biology techniques that have led to increased understanding of neurodegenerative disorders along with biologically based classifications of these disorders. Molecular diagnostics provide a powerful tool in the diagnosis of many neurological diseases. For example, genetic testing of mutations in disease-causing-genes has been leveraged to define and classify many of the heterogeneous inherited neurodegenerative syndromes (Gasser et al., 2001a,b, 2003). Changes in pathologies, biochemistries and genetics of patients can give us comprehensive information regarding the nature of a particular disease. However, molecular testing may be performed only after careful consideration and a genetic counseling. We describe in the following sections major neurologic disorders, and the ability and applications of molecular diagnostics in their detection.

## Alzheimer's disease

AD is the most common neurodegenerative disease in most Countries; it is a progressive, degenerative disorder that attacks the brain's neurons, resulting in loss of memory, thinking and language skills, and behavioral changes. Most of the neurons that degenerate in this disease communicate with other neurons using the chemical or neurotransmitter acetylcholine in the brain. Alterations in acetylcholinesterase (AChE) and butyrylcholinesterase (BuChE) can be observed in AD but acetylcholine depletion is the most common and striking of all symptoms (Rachakonda et al., 2004).

The two types of abnormal lesions that clog the brains of Alzheimer's patients are extracellular senile plaques (composed of beta-amyloid peptides) and intracellular neurofibrillary tangles (NFTs, composed of tau protein). Aβ forms as a result of enzymatic cleavage of the parent Amyloid Precursor Protein (APP). Proteases that are involved in the breakdown of APP, are α-, β-, and γ- secretases (Hardy and Selkoe, 2002; Rachakonda et al., 2004). The NFTs account for the synaptic degeneration or the atrophy of nerve cells following damage to the synaptically connected axons. NFTs are composed of paired helical filaments (PHF), which are principally made up of hyperphosphorylated insoluble form of tau protein (el-Agnaf and Irvine, 2002).

Diagnosis of AD is usually based on clinical observations and cognitive testing like neuropsychological testing which helps in the diagnosis and treatment of conditions like AD that affect emotion, thinking and behavior (Harvey, 2012). Neuropsychological tests accompany a comprehensive interview with the patient, and include tests to assess attention, memory, language, the ability to plan and reason, and modify behavior, as well as assessments of personality and emotional stability, that can also help the doctor and family better understand the impact of a disorder on a patient's everyday functioning. The disease is eventually confirmed by postmortem by demonstrating amyloid plaques and neurofibrillary tangles in the brain. However, the progression of the disease process is an ongoing phenomenon that significantly damages the brain long before clinical findings appear. Hence, molecular biologists, biomedical, and medicine experts and biotechnologists are coming together to design and develop advanced diagnostic molecular markers that might allow very early-stage diagnosis of AD and the objective assessment of its responses to putative treatments (Rachakonda et al., 2004). Several genetic and biochemical diagnostic biomarkers have been employed to detect and diagnose AD.

### Genetic biomarkers

Less than 5% of all cases of AD can be accounted for by mutations in the following three genes. Amongst them, mutations on the two homologous presenilin genes: presenilin 1 (*PS1*, MIM 104 311) located on chromosome 14, and presenilin 2 (*PS2*, MIM 600 759) located on chromosome 1, are most common and are responsible for over half of the known familial AD cases, whereas mutations in the gene for amyloid precursor protein (*APP*, MIM 104 760) located on chromosome 21) are comparatively less (Gasser et al., 2001a; Rachakonda et al., 2004). The presenilin genes code for proteins known as presenilin, which control the APP proteolysis into smaller peptides (Goodall et al., 2013). An abnormal increase in the activity of APP can be due to any missense mutation on one of these presenilin genes resulting into more Aβ peptides (Berezovska et al., 2003). The first genetic mutation linked to AD was found on the β*APP* gene (Rachakonda et al., 2004). This β*APP* gene, encodes a glycosylated trans-membrane protein which contains 770 amino acids in its longest isoform. This was confirmed by the fact that patients with Down's syndrome also developed similar plaques and suffered Alzheimer encephalopathy in their later years (Rachakonda et al., 2004). In addition to the mutations mentioned above, which can cause AD, the E4 allele of the *ApoE* is associated with the sporadic forms of AD (Bekris et al., 2010). Although E4 allele was detected in about 40–50% of all AD patients, but could not serve as a diagnostic marker based on the sensitivity criteria for biomarkers. Therefore, ApoE is regarded as a risk factor indicator rather than an actual genetic marker of AD. Along with positive family history, an early onset (in the 40s and 50s) which is common to all these monogenic forms, should act as an indication for molecular genetic diagnosis.

### Biochemical markers

The levels of tau protein and Aβ in cerebrospinal fluid (CSF) are the two most promising biochemical markers of AD. Aβ is secreted into the extracellular space and biological fluids, including CSF making Aβ42 a considerable indicator of AD (Sunderland et al., 2003). A decrease in levels of Aβ in CSF reflects AD and its sensitivity is around 80–90%. As AD progresses Aβ peptide from CSF aggregates to form plaques in the brain, thereby, lowering its concentration in the CSF. CSF-Aβ42 appears to be a remarkable biomarker for diagnosis of AD when used in combination with other AD biomarkers. CSF-tau also provides a very high sensitivity for AD but the reason for its abnormal increase in AD patients is not clear. The combination of both CSF-Aβ42 and CSF-tau may improve their specificity and sensitivity and can be an ideal biochemical marker set for AD (Sjögren et al., 2003). 2-(1-{6-[(2-[^18^F] Fluoroethyl) (methyl)amino]-2-naphthyl}ethylidene) malononitrile ([^18^F]FDDNP)-PET can determine the localization and load of neurofibrillary tangles and senile amyloid plaques in the human brain because of its ability to cross the Blood-Brain-Barrier (BBB). FDG-PET is used to measure the brain's energy utilization and to infer synaptic number (Wurtman, 2015). Therefore, PET Molecular imaging is employed to diagnose AD (Rachakonda et al., 2004; Sair et al., 2004). This discovery of a new binding site to Aβ_40_ fibrils as a result of FDDNP binding provides a new opportunity for early treatment of AD. The clinical sensitivity for AD using the “probable AD” category is 66 ± 17% relative to neuropathologically conformed diagnoses and can be increased to 90.5 ± 5.5%, by including “possible AD” patients at the expense of specificity. The sensitivity of 18F-FDG PET is 91 ± 3% (Bokde et al., 2011). The combined use of the [^18^F]FDDNP-PET molecular diagnostic labeling system and other diagnostic tests provide a new pathway to early diagnosis of AD (Sair et al., 2004; Wurtman, 2015). In a related work employing imaging spectroscopy, SPECT and PET tracers were used in the diagnosis and investigation of AD. Most tracers demonstrate the neuronal loss associated with the condition and results in regional decrease in glucose utilization which can be studied with ^18^F-FDG PET imaging (Young, 2009). SPECT and PET imaging are far more sensitive with the ability to detect tracers at 10^−9^–10^−11^ mol/L, at concentrations of tracers that will not disturb normal function (Bokde et al., 2011).

MicroRNAs (miRNAs) have been employed for early detection of AD (Grasso et al., 2014). miRNAs belong to a family of short, single-stranded 21–22 nucleotides-long non-coding RNAs that constitute about 1% of all human genes. They represent the most abundant class of small RNAs in animals. Further, miRNAs are found in high abundance within the nervous system, where they often replicate a brain-specific expression pattern and are usually found to be co-expressed with their targets. Their main roles are as key regulators of different biological functions including synaptic plasticity and neurogenesis, where they channelize the cellular physiology toward neuronal differentiation. Also, they can indirectly influence neurogenesis by regulating the proliferation and self-renewal of neural stem cells (Grasso et al., 2014).

It is interesting to note that miRNAs are deregulated in several neurodegenerative diseases, a spectrum of etiologies culminating in a final common pathway of neuronal cell death (Goodall et al., 2013). The dysfunction of miRNAs in neurodegenerative disorders can be leveraged for early diagnosis of AD, which is a novel approach to understanding neurodegenerative diseases (Goodall et al., 2013). Further, the use of miRNAs as biomolecular diagnostics markers has some advantages: first of all they allow ease of detection with extreme specificity. Furthermore, unlike large RNA molecules as mRNAs, miRNAs can be well preserved in formalin, paraffin embedded tissues (FFPE) and also in fresh snap-frozen specimens (Xi et al., 2007; Grasso et al., 2014).

Mild cognitive Impairment (MCI) is an intermediate state between normal aging and AD (and other dementias), which is usually defined as the first stage when clinical symptoms become evident (DeCarli, 2003). Plasma miRNA biomarkers were reported to detect MCI, where an initial pool of miRNAs was selected among known brain- and neuron-enriched miRNAs (Sheinerman et al., 2012). The researchers then identified biomolecular diagnostic marker pairs represented by two sets: the “miR−132 family” that consist of miR−128/miR−491−5p, miR−132/miR−491−5p and miR−874/miR−491−5p and the “miR−134 family” comprising miR−134/miR−370, miR−323−3p/miR−370 and miR−382/miR−370 with fairly high sensitivity and specificity at 79–100% and 79–95%, respectively. In a separate longitudinal study, the identified miRNA biomolecular diagnostic marker pairs successfully detected MCI in majority of patients at asymptomatic stage 1–5 years prior to clinical diagnosis (Sheinerman et al., 2012; Grasso et al., 2014).

Recent observations were made related to change in the levels of plasma phospholipids; that can be leveraged for developing new bimolecular diagnostic marker for AD. The reduction in the level of phospholipids is anticipated to enable the accurate prediction, that whether a cognitively normal individual is going to develop MCI or AD within 2 years (Wurtman, 2015). There is one study on blood-based biomarker panel for detecting preclinical AD with above 90% accuracy greater than that obtained from most published CSF studies (Mapstone et al., 2014).

## Parkinson's disease

The search for molecular diagnostics biomarkers in PD is critical to identify the disease in early stages which will allow monitoring the effectiveness of neuroprotective therapies for PD (Molochnikov et al., 2012). In PD, degeneration of neurons, more specifically dopaminergic neurons between the substantianigra (SN) and the striatum occur. As a result, a great majority of dopamine producing cells in the substantianigra are lost in patients with PD. The symptoms of PD are trembling in hands, arms, legs, and face; stiffness of the limbs and trunk; slowness of movement; and impaired balance and coordination. As these neurons are progressively destroyed, patients may have difficulty walking, talking and completing other simple tasks (Rachakonda et al., 2004). PD usually affects people over the age of sixty.

Currently, only clinical criteria are employed to diagnose PD (Molochnikov et al., 2012). The evaluation of the clinical status and evolution of PD are based on various factors and medical steps. These include examination of symptoms, utilizing structured scoring systems [Unified Parkinson's Disease Rating Scale, (UPDRS), Short Parkinson Evaluation Scale, (SPES), Scales for Outcomes in Parkinson's diseases—(SCOPA), and the Hoehn and Yahr (H&Y) staging scale; (Molochnikov et al., 2012)]. Clinical criteria based diagnosis of PD can be done with a typical presentation and positive response to levodopa with a sensitivity of 93%. However, the major limitation of this technique is differential diagnosis from other entities presenting Parkinsonism [e.g., essential tremor, progressive supranuclear palsy (PSP), multisystem atrophy (MSA), corticobasal degeneration (CBD)] that may be challenging.

Recent research has shown that molecular diagnostic tools can be leveraged to overcome the current challenges on limitations to early detection and effective differential diagnosis. A molecular diagnostic signature in blood that identifies early PD was reported. An assessment was done on whether a gene signature could be detected in blood from early/mild PD patients that could support the diagnosis of early PD, focusing on genes found particularly altered in the substantianigra of sporadic PD (Molochnikov et al., 2012). The research findings provide evidence on the ability of a five-gene panel to diagnose early/mild PD, with a possible diagnostic value for detection of asymptomatic PD before overt expression of the disorder (Molochnikov et al., 2012).

This pilot study demonstrated that the blood gene model can have strong predictive value for PD diagnosis that possibly may help to identify individuals at presymptomatic stages (patients with depression, sleep disturbances or hyposmia or patients carrying genetic risk factors) that are good candidates for neuroprotective treatment. Such a biomolecular diagnostic marker for PD can be of tremendous value for the identification of a pathophysiological subgroup of PD patients that may respond favorably to agents targeting the mechanisms reflected by the gene panel.

### Genetic biomarkers

PD is inherited in a Mendelian autosomal dominant or autosomal recessive fashion in a small number of families. Mutations were found in α-synuclein (*SNCA*) and leucine-rich repeat kinase2 (*LRRK2*) genes for late-onset disease and parkin (*PARK2*), ubiquitin carboxy-terminal hydrolase L1 (*UCH-L1*), PTEN Induced Putative Kinase1 (*PINK1*), oncogene DJ1 (*DJ1*) for early onset (Grasso et al., 2014).

Point mutations, duplications, and triplication in the α-synuclein gene, which is located on chromosome 4, are a characteristic of PD and they occur in most forms including the rare early onset familial form of PD. Genes and gene products have been identified by characterizing the monogenetic autosomal dominant forms of PD. Several gene products of the mutated genes in the autosomal dominant forms have been linked to mitochondrial dysfunction, oxidative stress, and mishandling of impaired or aberrant forms of the gene products (e.g., oligometric α-synuclein) (Miller and O'Callaghan, 2015). More than 70 mutations on the large parkin gene have been associated with the early-onset form of Parkinsonism. Mutations in the parkin gene may account for PD in as many as 50% of familial cases of autosomal recessive juvenile Parkinsonism (Pankratz et al., 2003). Another gene ubiquitin carboxy-terminal hydrolase L1 (*UCH-L1*) located on chromosome 4 encodes a protein which belongs to the family of deubiquitinating enzymes. Protein UCH-L1 constitutes 1% of brain protein and its function is presumed to act to recycle ubiquitin by hydrolyzing the ubiquitinated peptides. This enzyme plays a role in modifying the damaged proteins that might otherwise accumulate to toxic levels in the neuron (Leroy et al., 1998). Also two homozygous mutations in *PINK1* gene associated with PD were found in Spanish and Italian families. This finding provided additional evidence that *PINK1* mutations are associated with *PARK6* (Valente et al., 2004). And the mutations associated with *PARK7* are in *DJ-1* gene (Bonifati et al., 2003). Evidence suggests DJ-1 protein involvement in oxidative stress and neurodegeneration. Slow progression of symptoms with sustained response to levodopa treatment is the clinical characteristics of DJ-1 Parkinsonism (Dekker et al., 2003). Revealing the physiological role of these genes may promote the understanding of the mechanisms of brain neuronal maintenance.

### Biochemical markers

Two other major biomolecular diagnostic markers have been employed to recognize the onset of PD. They include (1) the loss of the dopamine transporter “DAT” detected by PET imaging and (2) the presence of the α-synuclein protein located in the Lewy body lesions. DAT mediates uptake of dopamine (DA) into dopaminergic neurons by an electrogenic, Na^+^- and Cl^−^-transport-coupled mechanism. DA and cocaine (uptake blockers) would bind to both the shared and separate domains on the transporter, which is observed to be dramatically influenced by the presence of cations. DAT is also involved in the uptake of toxins generating Parkinson's syndrome. Thus, the localization of striatal, preferentially putamen DAT concentration is considered a high sensitivity parameter for the detection of early phases of PD and best molecular diagnostic marker (Marek et al., 2000; Rachakonda et al., 2004; Shinto et al., 2014). Discovery of Lewy bodies and Lewy neuritis, the characteristic lesions in brains of patients with PD and dementia is due to two mutations in α-synuclein gene. Given that α-synuclein is also found in other synucleinopathies, it should be used with the aid of other diagnostic methods to increase the specificity and sensitivity for PD (Duyckaerts and Hauw, 2003).

Several imaging techniques have been employed for the diagnosis of PD, for example, PET with [^18^F]-Dopa tracer (Loane and Politis, 2011) and single photon emission tomography (SPECT) with [123I]-β-CIT (Tissingh et al., 1997). PET is considered to be the most useful tool for PD diagnosis by measuring the emission of positrons from the brain after a small amount of radioactive isotopes or tracers have been injected into the blood stream. Studies have shown a mean reduction of 40% in striatal 18F-Dopa uptake between controls and patients with PD (Bokde et al., 2011). PD patients related to medication typically have normal 18F-Dopa distribution. Tracer [^18^F]-Dopa has very limited clinical availability but Ioflupane Iodine-123 (DatSCAN) is a widely available SPECT tracer which models the presynaptic dopamine receptor (DaT) system. SPECT only differ to PET in that it uses isotopes with longer half-lives that can be stored on site (Rachakonda et al., 2004; Young, 2009). Recently a SPECT imaging with ^99m^Tc-TRODAT-1 was conducted in 16 consecutive PD patients (9 men; 7 women) and in 6 age matched healthy volunteers (4 men; 2 women; Shinto et al., 2014). The images were obtained 3 h after the intra-venous injection of the tracer. A stepwise reduction in specific striatal uptake of ^99m^Tc-TRODAT-1 was found with increasing disease severity between healthy controls vs. Stage I vs. Stage II vs. Stage III in PD patients (i.e., 3.77 vs. 2.56 vs. 1.57 vs. 0.63, *P* < 0.05). ^99m^Tc-TRODAT-1 is accurate and widely available for the assessment of DAT activity. These techniques could improve differential diagnosis of Parkinsonism, but cost-effectiveness remains a problem (Jankovic et al., 2000; Molochnikov et al., 2012).

Biological fluids are excellent source for biomarkers as their close proximity to cells reflects their biological condition and are simple to obtain and cost-effective (Shinde et al., 2015). With the increasing relevance of miRNAs in biofluids the development of circulating biomarkers for PD has great potential. A study using qRT-PCR suggested that in peripheral blood the expression levels of miR-1, miR-22-5p, and miR-29 allow to distinguish PD patients from healthy subjects, and also miR-16-2-3p, miR-26a-2-3p, and miR30a differentiate between treated and untreated patients (Margis et al., 2011). In a recent study using next generation sequencing for total blood leukocytes it was found that, 16 miRNAs including miR-16, miE-20a and miR-320 significantly altered in PD patients compared to healthy controls (Soreq et al., 2013; Grasso et al., 2014).

There is no standard diagnostic test for Parkinson's. Researchers are still working to develop an accurate “biological marker,” such as a blood test or an imaging scan. To date, tests consist of specialized brain scanning techniques to measure the dopamine system and brain metabolism is the best objective test for PD (Torrent et al., 2015). But these tests are expensive and performed only in specialized imaging centers.

## Amyotrophic lateral sclerosis

**Figure 1:**
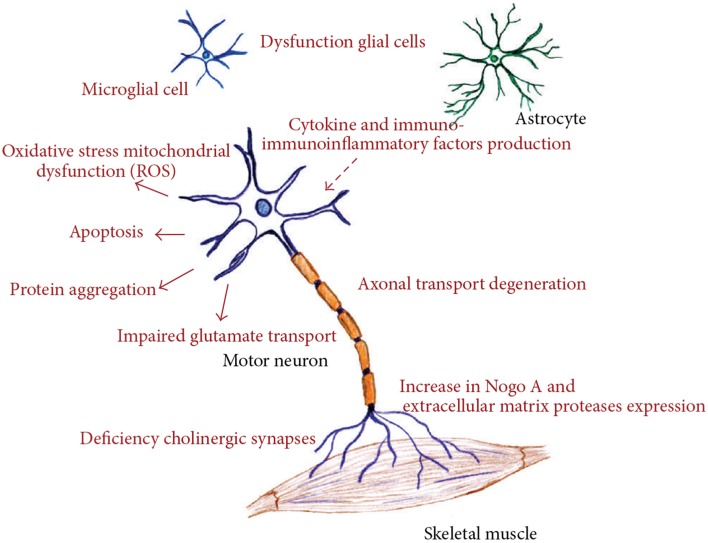
**Different molecular pathways altered in amyotrophic lateral sclerosis, all of these deregulated mechanisms prompt motor neuron death. (Figure taken from: Calvo et al., 2014)**.

ALS is a rapidly progressive, invariably fatal neurological disease that attacks the neurons responsible for controlling voluntary muscles. Messages from motor neurons in the brain (called upper motor neurons) are transmitted to motor neurons in the spinal cord (called lower motor neurons) and from them to particular muscles. In ALS, both the upper and lower motor neurons degenerate or die, and stop sending messages to muscles. Unable to function, the muscles gradually weaken, waste away (atrophy), and have very fine twitches (called fasciculation). Eventually, the ability of the brain to start and control voluntary movement is lost. ALS is a result of complex array of factors, including all or just some of these like oxidative stress, endoplasmic reticulum stress, mitochondrial dysfunction, dysregulated endosomal trafficking, dysregulated transcription, and RNA processing, excitotoxicity, apoptosis, inflammation, and genetic susceptibility (Figure 1; Calvo et al., 2014).

The diagnosis of ALS is primarily based on the symptoms, clinical examination, and a series of tests to rule out other diseases. We do not have a practical diagnostic biomarker for ALS in spite of intensive research over the past several years, which lead to diagnostic delays. Neurophysiological approaches like motor unit number estimation (MUNE) and electromyography (EMG), a special recording technique that detects electrical activity in muscles, routinely play a key role in search of valid biomarkers to recognize ALS (DeJesus-Hernandez et al., 2011; Joyce and Carter, 2013).

### Genetic biomarkers

Mutations in more than a dozen genes have been found to cause familial ALS. About one-third of all familial cases (and a small percentage of sporadic cases) results from a defect in a gene known as “chromosome 9 open reading frame 72,” or *C9orf72*. The function of this gene is still unknown. Another 20 percent of familial cases result from mutations in the gene *SOD1* that encodes the enzyme copper-zinc superoxide dismutase 1 (SOD1). Although it is still not clear how mutations in the SOD1 gene lead to motor neuron degeneration, there is increasing evidence that mutant SOD1 protein can become toxic. Another 5 percent of familial cases and < 1% sporadic cases results from mutations in the gene *FUS*, which encodes fused in sarcoma/translocated in liposarcoma (FUS/TLS), and three percent of the remaining familial cases result from mutations in *TARDBP* gene, which encodes TAR DNA binding protein-43 (TDP-43; Robelin and Gonzalez De Aguilar, 2014).

Another possibility is the role of epigenetics. Epigenetic mechanisms modify chromatin structure and can mimic genetic change; these modifications are reversible, heritable, and non-heritable in DNA or chromatin structure, but not in DNA sequence (Martin and Wong, 2013). There are different types of epigenetic modifications, such as DNA methylation and histone acetylation. In a study, CpG methylation microarrays were used to profile DNA extracts of postmortem spinal cord from sporadic ALS cases (Figueroa-Romero et al., 2012). Bisulfite-converted DNA was amplified and hybridized to Infinium Human Methylation27 DNA BeadChip arrays. Methylation of 27,578 CpG sites spanning 14,495 human genes was determined and hypo- or hypermethylation 726 Martin and Wong was found in 112 genes in ALS cases (Figueroa-Romero et al., 2012).

### Biochemical markers

Studies showed that the expression of the metabotropic glutamate receptor subtype mGLUR2, which is known to provide protection against excitotoxicity, was diminished in ALS T lymphocytes (Poulopoulou et al., 2005), which was confirmed by the high concentration of glutamate detected in the CSF of many patients. The enzymatic activity of glutathione peroxidase and SOD1 was also found to decrease in ALS erythrocytes (Cova et al., 2010). Increased amounts of 8-hydroxy-2′-deoxyguanosine (8OH2′dG in ALS patients, which is a product of the oxidative injury to DNA is also a good biomarker (Bogdanov et al., 2000). There is an uncontrolled increase of microglial cells in the central nervous system and other immune cells, contributing to motor neuron degeneration in ALS (Philips and Robberecht, 2011). The factors involved in these inflammatory reactions like cytokines can be followed in the periphery as potential biomarkers. Therefore, the circulating levels of interleukin-6, tumor necrosis factor-α (TNF-α), interferon-γ, monocyte chemo attractant protein-1 (MCP-1), and wide-range C-reactive protein (wrCRP) were found to be increased in ALS patients (Robelin and Gonzalez De Aguilar, 2014). Neurite outgrowth inhibitor (Nogo), one of the potential biomarkers for ALS possesses axonal growth inhibitory activity and has a central role in ALS (Fergani et al., 2005). Together with the absence of reliable and powerful diagnostic and prognostic biomarkers, ALS is a major cause for concern.

## Huntington's disease

HD is a heritable neurodegenerative disorder that can affect motor, cognitive and psychiatric functioning. Decline of cognitive ability and change in personality are symptoms of HD (Mastrokolias et al., 2015). The pathology is caused by an expanded CAG repeat in the *HTT* gene, resulting in a mutant huntingtin protein (mHTT). Mutant protein aggregate formation and neuronal cell loss, with transcriptional deregulation are prominent feature of HD brain tissue (Runne et al., 2008). Recently, mutant huntingtin protein (mHTT) levels were quantified by an ultrasensitive single-molecule counting (SMC) mHTT immunoassay for the first time in CSF samples of individuals bearing HD mutation (Wild et al., 2015). It is important to have a disease progression biomarker that should be able to identify changes before clinical symptoms. Huntingtin is ubiquitously expressed and mutant huntingtin-specific changes could be reflected by gene expression changes in blood. Involvement of leukocytes in immune system regulation made blood an ideal source for identifying HD events such as peripheral inflammation. Several studies have also identified HD blood mRNA changes using microarray technology, but were difficult to validate across studies (Lovrecic et al., 2009). The validation of biomarkers for HD has been always challenging as the disease present itself through a variety of symptoms and progression rates.

### Molecular biomarkers

Individuals with HD over expressed the gene, H2A histone family, member Y (*H2AFY*), in their blood (Hu et al., 2011). The overexpression of this gene in both the blood and the brain was validated in samples from clinical studies. Specifically, the research demonstrates a 1.6-fold overexpression of *H2AFY* in patients with HD. Recently a study showed gene expression profiling, with the help of next-generation sequencing and Fluidigm technologies and yielded a set of five genes as a potential HD biomarkers that are highly expressed in HD blood (Mastrokolias et al., 2015). Prokineticin 2 (*PROK2*) has been proposed to have a role in the circadian rhythms alterations that have been shown to correlate with cognitive impairment in HD (Aziz et al., 2010). Pharmacological imposition of sleep slows cognitive decline and reverses deregulation of *PROK2* in HD models. *PROK2* is very promising biomarker of HD progression. Evidences suggest that gene repression mechanisms are also associated with HD and Zinc finger protein 238 (*ZNF238*) is a transcriptional repressor which is involved in brain development and myogenesis (Zhai et al., 2005). A recent gene expression study showed that the increase in mRNA levels of Aquaporin 9 (*AQP9*) and presence of *AQP9* in blood could represent peripheral or central inflammatory events when accompanied with increase in levels of four other genes (Mesko et al., 2010). Annexin A3 (*ANXA3*) and Cysteine-rich, transmembrane (TM) module (*CYSTM*) are two other potential biomarkers for HD (Borovecki et al., 2005; Venancio and Aravind, 2010). *ANXA3* is found to be up regulated in neuronal injury models and *CYSTM* is involved in stress response specifically heavy metal tolerance.

### Biochemical markers

Variability in clinical phenotype of HD and potential confounds of environmental and pharmacological factors, results in the use of combination of different biomarkers that might be efficient in tracking the progression of HD. Many potential biomarkers have been identified during the discovery of disrupted homoeostasis in HD. In a recent study, with the help of cross-sectional MRS the researchers have distinguished putaminal metabolites in pre-manifest and early HD individuals from controls (Sturrock et al., 2015). It was found that the total N-acetyl aspartate (tNAA) is lower in early HD and pre-manifest HD than in controls whereas the gliosis marker myo-inositol (MI) was robustly elevated in early HD. Another study, have also demonstrated metabolite changes in the caudate nucleus and putamen of HD gene carriers around disease onset (van Den Bogaard et al., 2014). These correlations of Total NAA with disease burden score suggest that this metabolite may be useful in identifying neurochemical responses to therapeutic agents.

Vasopressin has a role in fluid balance homoeostasis, increase in serum concentrations of vasopressin have been reported in HD (Wood et al., 2008). Increased concentrations of 8-hydroxy-2-deoxyguanosine (8OHdG), an indicator of oxidative DNA injury, and increased concentrations of plasma lipid peroxide, lactic acid, 4-hydroxynoneal, and malondialdehyde in patients with HD make them a potential biomarker (Weir et al., 2011). Decrease in glutathione peroxidase and copper–zinc superoxide dismutase was observed in erythrocytes from HD patients compared with controls (Chen et al., 2007). Elevated cytokines levels including interleukins 4, 6, 8, 10, and 23, TNF-α, and clusterin have been identified in the post-mortem brain and plasma samples of patients with HD (Dalrymple et al., 2007). The inflammatory profile differences between control and gene carriers serve as potential biochemical marker for HD including rest of the above biomarkers. All these biomarkers would facilitate accurate evaluation of the effectiveness of new therapies and improve the safety and efficiency of clinical trials.

## Conclusion and future perspective

In this short review, we have described current trends in the applications of molecular diagnostic techniques for early detection and diagnosis of neurodegenerative disorders focusing on AD, PD, ALS, and HD. We have discussed several biomolecular diagnostic markers that have been identified in the past decade that have an enormous scope for further research in the areas of both genetic and biochemical molecular markers. Biomolecular diagnostic markers may provide new insights regarding different diagnosis and therapeutic guidance to specific neurodegenerative diseases.

Molecular diagnostics for neurodegenerative diseases represent a multidisciplinary research area where a robust collaboration between neurologists, psychologists, biologist, and biomaterials scientists and other trained personnel with the necessary experience in managing the diseases is required. Future research directions might include designing and developing a combination of several biomolecular diagnostic markers for multifunctionalities. Such a multifunctional molecular diagnostic technology platform would significantly enhance the accuracy, specificity and sensitivity. Developing molecular diagnostics based on circulating miRNAs could also be a highly promising approach for developing minimally invasive screening tests for neurodegenerative disorders.

Future studies may also include developing a multicenter and prospective design of molecular diagnostics tools; measurement of multiple potential biomarkers and a prolonged clinical follow-up period (till death as end-point) that provide assessment of both clinical features and determinations of the biological diagnostics and eventually neuropathological confirmation by examining the brains of patients at death.

### Conflict of interest statement

The authors declare that the research was conducted in the absence of any commercial or financial relationships that could be construed as a potential conflict of interest.
